# Consequences of waterlogging in cotton and opportunities for mitigation of yield losses

**DOI:** 10.1093/aobpla/plv080

**Published:** 2015-07-17

**Authors:** Ullah Najeeb, Michael P. Bange, Daniel K. Y. Tan, Brian J. Atwell

**Affiliations:** 1 Department of Plant and Food Sciences, Faculty of Agriculture and Environment, The University of Sydney, NSW 2015, Australia; 2 CSIRO Agriculture Flagship, Australian Cotton Research Institute, Narrabri, NSW 2390, Australia; 3 Department of Biological Sciences, Faculty of Science, Macquarie University, Sydney, NSW 2109, Australia

**Keywords:** Cotton, ethylene, fermentation, hypoxia, photosynthesis, waterlogging

## Abstract

Climatic variability, typified by erratic heavy-rainfall events, causes waterlogging in intensively irrigated crops and is exacerbated under warm temperature regimes on soils with poor internal drainage. Irrigated cotton is often grown in precisely these conditions, exposing it to waterlogging-induced yield losses after substantial summer rainfall. This calls for a deeper understanding of mechanisms of waterlogging tolerance and its relevance to cotton. Hence this review suggests possible causes of waterlogging-induced yield loss in cotton and approaches to improvement of waterlogging tolerance, drawing upon the slight body of published data in cotton and principles from other species. The yield penalty depends on soil type, phenological stage and cumulative period of root exposure to air-filled porosities below 10 %. Events in the soil include O_2_ deficiency in the root zone that changes the redox state of nutrients, making them unavailable (e.g. nitrogen) or potentially toxic for plants. Furthermore, root-derived hormones that are transported in the xylem have long been associated with oxygen deficits. These belowground effects (impaired root growth, nutrient uptake and transport, hormonal signalling) affect the shoots, interfering with canopy development, photosynthesis and radiation-use efficiency. Compared with the more waterlogging-tolerant cereals, cotton does not have identified adaptations to waterlogging in the root zone, forming no conspicuous root aerenchyma and having low fermentative activity. We speculate that these factors contribute substantially to the sensitivity of cotton to sustained periods of waterlogging. We discuss the impact of these belowground factors on shoot performance, photosynthesis and yield components. Management practices, i.e. soil aeration, scheduling irrigation and fertilizer application, can reduce waterlogging-induced damage. Limiting ethylene biosynthesis using anti-ethylene agents and down-regulating expression of genes controlling ethylene biosynthesis are strong candidates to minimize yield losses in waterlogged cotton crops. Other key pathways of anoxia tolerance are also cited as potential tools towards waterlogging-tolerant cotton genotypes.

## Abstract (precis)

Cotton is a major world crop that is notoriously susceptible to waterlogging damage, particularly when cultivated on fine-textured soils. However, damage is also exacerbated because of inadequate acclimation of roots to low oxygen levels, and secondary effects on shoots. Despite the commercial importance of cotton, very little has been published when compared with waterlogged cereals. This review provides a comprehensive view of the constraints on cotton in low-oxygen conditions, including absence of aerenchyma and the inadequacy of fermentation to overcome waterlogging damage. We emphasise the possibilities of improved tolerance through management practices, manipulation of hormone pathways and gene technologies to modify perception and response to low-oxygen environments.

## Introduction

Waterlogging is a worldwide phenomenon that strongly influences the distribution of plant species and crop production. According to a 2007 FAO report, 20–30 million hectares of irrigated land area was affected by soil waterlogging as a result of poor soil drainage, intensive irrigation and highly variable weather patterns. This in turn affects crop production in many parts of the world (Setter and Waters 2003). Soil waterlogging dramatically reduces the oxygen (O_2_) diffusion rate through soils, and when coupled with O_2_ depletion by respiration of microorganisms and plant roots, soil O_2_ levels quickly fall below critical levels. This process is further exacerbated by high temperatures, which accelerate respiratory activity. Even under incomplete waterlogging when soil air-filled porosity might only be marginally below 10 % (Hodgson 1982), these levels can be critical for roots, lowering respiration rates below the level required to sustain maximum energy production.

Submerged plant organs undergo a dramatic decline in O_2_ availability. Plant species which are adapted to low O_2_ conditions often obtain atmospheric O_2_ through rapid diffusion along gas-filled root aerenchyma (air spaces in cortical tissues). In such cases, cellular O_2_ deficiency not only depends on O_2_ concentrations in the bulk soil but also on the length of diffusion path, resistance to radial leakage from roots, respiration rate of root tissues and thickness of the diffusive boundary layer around roots (Armstrong *et al*. 2009). Once O_2_ concentrations in root tissues drop below the critical O_2_ pressure (COP) for respiration, they become O_2_ limited (Armstrong and Drew 2002). In roots, partial O_2_ deficiency in soils (hypoxia) can result in the complete absence of O_2_ (anoxia) in the stele, inhibiting aerobic respiration, energy generation and nutrient acquisition (Jackson and Drew 1984).

Oxygen deficiency initiates various deleterious events, viz. metabolic pathways that cause accumulation of by-products of fermentation in roots (e.g. acetaldehyde, ethanol), acid loads in cells (Felle 2005) and toxic substances in soil (volatile fatty acids, phenolic acid, hydrogen sulfide, nitric oxide (NO), methane and carbon dioxide (CO_2_)) (Zeng *et al*. 2013). Waterlogging alters the cation exchange capacity of soil particles and valency of nutrients (more reduced forms), making them toxic or unavailable for plant uptake (Setter *et al*. 2009). Hypoxia-induced nutrient deficiency/toxicity interferes with a range of shoot physiological processes such as photosynthesis, respiration and growth, causing chlorosis and necrosis and ultimately, plant death (Dodd *et al*. 2013; Bailey-Serres and Colmer 2014).

Waterlogging tolerance in plants is a function of tolerance to anaerobiosis and chemical toxicities (Setter *et al*. 2009). Plants undergo various anatomical, morphological, physiological and metabolic adjustments for their survival in O_2_-deficient environments, although rates of acclimation vary with species, temperature and rapidity of the onset of waterlogging. Development of aerenchyma is a common but not universal response to flooding, occurring particularly in grasses where it facilitates O_2_ diffusion along the axes of roots (Jackson and Drew 1984). While this important phenomenon has been exhaustively studied in monocotyledons and marsh species, few data are available for dicotyledonous crop species.

Some genera of dicotyledons (e.g. *Rumex* and *Lotus*) have been shown to express waterlogging tolerance via a suite of morphological changes. The range of mechanisms include increased root porosity (intercellular spaces), development of adventitious root and hypertrophied lenticels and rapid shoot elongation during submergence (Kozlowski and Pallardy 1984; Teakle *et al*. 2007; Bailey-Serres and Voesenek 2008).

At the physiological level, waterlogging may induce stomatal closure, thereby decreasing transpiration and photosynthesis in a variety of plant species. Metabolic responses including energy production via fermentation, catalytic adjustments, anaerobic protein synthesis and hormonal regulation are also crucial for survival of plants exposed to low O_2_ concentration.

Cotton (genus *Gossypium*) belongs to family Malvaceae. Although, there is debate over taxonomy of the genus *Gossypium*, Smith (1995) classified 43 species of *Gossypium*, of which 37 are diploid (2*n* = 2*x* = 26) and six are tetraploid (2*n* = 4*x* = 52). On the basis of genetic similarity, this genus is divided into eight diploid genomes (designated A–G and K) and one tetraploid genome (Stewart 1995). At present, *Gossypium hirsutum* L. and *Gossypium barbadense* L. are the major cultivated cotton species, both being AD-genome tetraploid species (Wendel 1989). *Gossypium hirsutum* contributes up to 90 % of the world fibre production (Jenkins 2003) while 5 % comes from *G. barbadense* (Wu *et al*. 2005).

**Figure 1.**
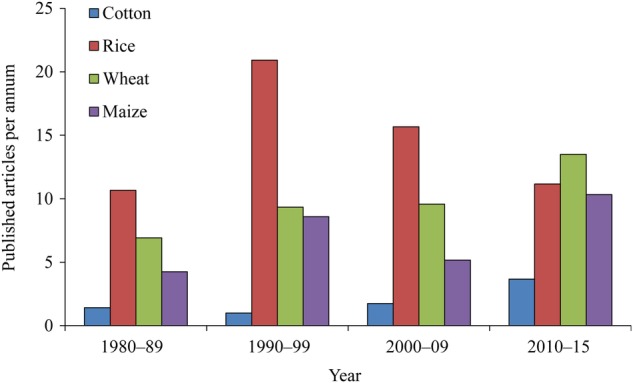
Annual publication rate for manuscripts dealing with waterlogging, anaerobiosis, anoxia and/or O_2_ deficiency in cotton and other crop species (rice, maize and wheat).

Cotton (*G. hirsutum*) is an important fibre and oilseed crop grown over 30 million hectares worldwide (USDA 2012). Improvements in production systems and breeding programs over the past decade have substantially increased the per hectare cotton lint yield (International Cotton Advisory Committee on Cotton Yields—ICAC 2009). However, unfavourable environments significantly inhibit cotton production. In particular, cotton is frequently cultivated in poorly drained heavy clay soils that may incur significant yield penalties after heavy summer rainfall events that cause subsequent waterlogging. A better understanding of physiological and biochemical responses to hypoxia/anoxia could help to improve tolerance through improved soil monitoring and selective plant breeding. This review aims to provide information on the possible mechanisms through which waterlogging damages cotton crops and suggests remediation pathways. However, much of the analysis that follows is based on inferences from studies on waterlogging damage in other crop species because there has been relatively little investigation of cotton under waterlogging in the past 35 years (Fig. 1).

## Root Growth

Sustained elongation of roots, even in intensively irrigated and fertilized crops, is critical for resource acquisition during vegetative growth. Therefore, the environmental factors that influence root growth, such as waterlogging, are critical if final yield is to be maximized.

Laboratory studies show that root apices must be at or above the COP for normal root growth and extension (Armstrong and Webb 1985); COP varies among plant species. The O_2_ concentration below which root extension begins to decline depends on the COP for respiration (COP_R_), which in turn is influenced by the characteristics of the tissues through which O_2_ must diffuse (e.g. proportion of stele) and the O_2_ affinity of oxidases (Armstrong and Drew 2002). In field-grown cotton, root growth is a function of O_2_ consumption in the soil by roots and microbes (Meyer *et al*. 1987); growth inhibition starts under mildly hypoxic (O_2_ < 10 %) conditions. Exposure of cotton plants to short-term (2–3 min) anoxia caused transitory cessation of tap root elongation but it resumed as the O_2_ supply was re-established, while just 3 h of anoxia resulted in complete death of the terminal apices of cotton roots (Huck 1970). We consider that cotton roots are relatively intolerant to low O_2_ supply.

However, the processes responsible for slow root extension in waterlogged soils are complex, with the primary impact of respiratory impairment interacting with a plethora of downstream (secondary) effects. Armstrong and Drew (2002) proposed that inhibited energy generation in hypoxic root tips arrests root extension by inhibiting cell division with consequences for water and nutrient acquisition. Zhang *et al*. (2015) also demonstrated that despite up-regulation of fermentative genes, waterlogging also induces oxidative damage to cotton root tissues.

In order for sufficient adenosine triphosphate (ATP) turnover to be sustained by fermentation during O_2_ deficits, well-adapted plant tissues can accelerate carbohydrate breakdown and therefore, energy generation from glycolytic flux (Gibbs and Greenway 2003). This heightened consumption of carbohydrates can cause carbohydrate starvation, a situation that is exacerbated when translocation of carbohydrates from leaves to roots is suppressed (Brändle 1991) and sugar unloading in roots is impaired (Saglio 1985). There are more subtle measures that conserve energy in anoxia-tolerant tissues, with strong arguments being made for the re-direction of scarce ATP to critical reactions (Edwards *et al*. 2012).

### Root structural modification

A comprehensive study of waterlogging tolerance using different plant species confirmed that primary tolerance mechanisms reside in roots rather than in shoots (Davies *et al*. 2000). Specifically, the root system plays a central role in shoot response to waterlogging through:

- Water and nutrient uptake from soils and supply to the aboveground organs;
- Synthesis of hormones regulating plant response to hypoxia.

Root structural characteristics and functional processes strongly depend on biotic and abiotic soil factors, and are especially strongly influenced by the distribution and availability of gases and nutrients in waterlogged soil. The major pathways for O_2_ supply to roots are through the soil medium or through intercellular gas spaces and aerenchyma when they exist in the shoot–root continuum. In waterlogged or O_2_-deficient soils, shoots and their interface with the atmosphere become the major source of O_2_ supply to roots of flood-tolerant species. Depending on the shape and arrangement of cortical cells, path lengths, cellular O_2_ demands and radial losses, radius of the stele vs cortex and shape of the root apex, roots will receive some proportion of the O_2_ they require for normal aerobic function (Colmer 2003). Within a single root axis, apices and the stele are potentially anoxic while the outer cortical tissues may continue to be aerobic (Armstrong and Beckett 1987). Factors controlling these tissue-specific variations in O_2_ status are not well described for less tolerant dicotyledonous species such as cotton, where phenotypic variation in radial dimensions and biophysical characteristics of roots might yet be exploited.

**Figure 2.**
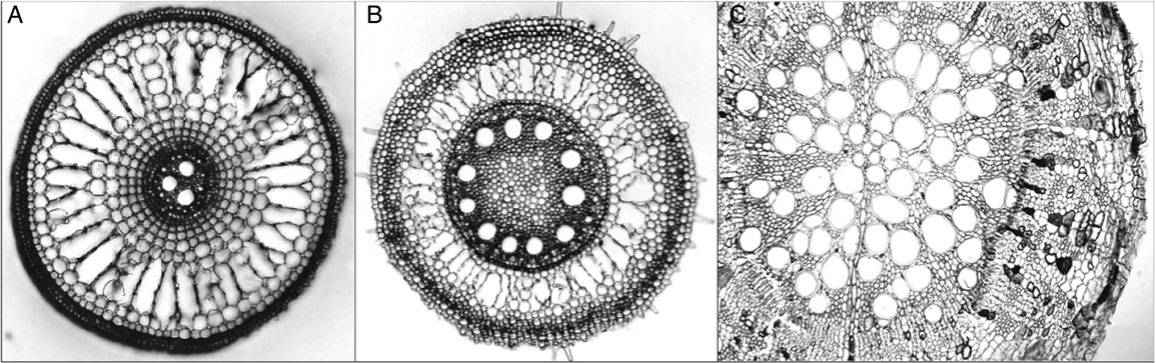
Waterlogging-induced aerenchyma formation in roots of rice (A) and wheat (B) (micrographs courtesy of *Plants in Action*; Atwell *et al.* 1999), while no aerenchyma formation in waterlogged cotton roots (C) where cortical cells are densely packed (Conaty *et al*. 2008).

**Figure 3.**
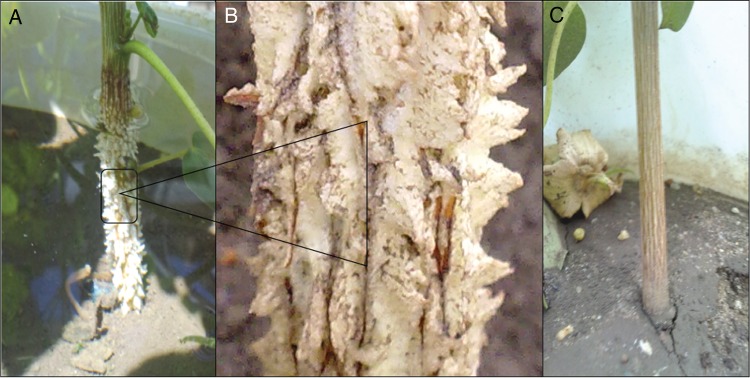
Development of hypertrophic lenticels at the base of cotton stems under long-term waterlogging. (A) Stem of waterlogged cotton; (B) magnified view of waterlogged cotton stem; (C) cotton stem under non-waterlogged conditions.

Notwithstanding these adaptive features, primary root elongation, even in waterlogging-tolerant plants, is suppressed when exposed to O_2_ deficiency. Tolerant species such as many grasses develop lateral and adventitious roots, enabling nutrient uptake from waterlogged soils. When cotton plants were re-aerated, primary axes initiate lateral roots after death of the apical meristem. Initiation of adventitious root primordia is controlled by an interaction between plant hormones, particularly ethylene (Verstraeten *et al*. 2014). Ethylene accumulation also triggers various adaptive traits within root axes, such as cortical cell senescence, increased fractional root porosity and secondary growth of phelloderm in dicotyledonous species (Evans 2004). Such changes facilitate gaseous exchange between aerobic shoots and anaerobic roots of various crops including wheat, maize and rice (Armstrong and Drew 2002). Significantly, eudicotyledons such as cotton do not display the same widespread tendency to form aerenchymatous roots (Fig. 2) (Conaty *et al*. 2008). However, there are other potential adaptations to waterlogging, with cotton enhancing survival in short-term hypoxia by developing hypertrophic lenticels (Fig. 3); similar responses have been reported in *Lotus tenuis* (Teakle *et al*. 2007).

### Nutrient uptake

The acquisition of inorganic nutrients is critical for high productivity of irrigated crops such as cotton. A fall in O_2_ levels after heavy rainfall initiates a series of chemical reactions within the soil. As the intensity of waterlogging increases, soils shift from hypoxic to anoxic and slowly, redox potentials enter the range that renders ions toxic or unavailable. Excluding, sequestering or re-oxidizing these solutes is important to avoid root damage. These control mechanisms depend heavily upon rhizosphere O_2_ levels if re-oxidation of toxic ions is to be achieved, but this is unlikely in non-aerenchymatous cotton roots until the bulk soil begins to re-aerate. Where atmospheric O_2_ supply is very limited such as the case in cotton, root energy status to sustain active transport systems and membrane integrity become critical, both during and after waterlogging events. Evidence suggests that a combination of these adaptive mechanisms can prevent Mn toxicity from developing after 8 days of waterlogging (Hocking *et al*. 1987). However, damage to root tissues, particularly apices, is not unique to periods of O_2_ deprivation, with re-aeration post-waterlogging imposing a new set of challenges for roots as reactive oxygen species impair metabolic processes (Blokhina *et al*. 2001; Shabala *et al*. 2014).

Because hypoxia impedes root ATP synthesis, it alters the activity of plasma membrane H^+^-ATPases (Jackson *et al*. 2003). Since uptake of mineral nutrients such as N, P, K, Mg and Ca is generally energy-dependent (Marschner and Marschner 2011), partial membrane depolarization and reduced ATP availability for pumps suppress their uptake (Steffens *et al*. 2005). Colmer and Greenway (2011) proposed that as roots become reliant on O_2_ supply from shoots, nutrient uptake from soils may continue into root hypoxic epidermal and cortical cells. However, development of an anoxic stele inhibits energy-dependent ion transport into the xylem. In such cases, small quantities of ions could still pass to the xylem tissues via plasmodesmata and non-selective outward-rectifying channels (Pang and Shabala 2010).

**Figure 4.**
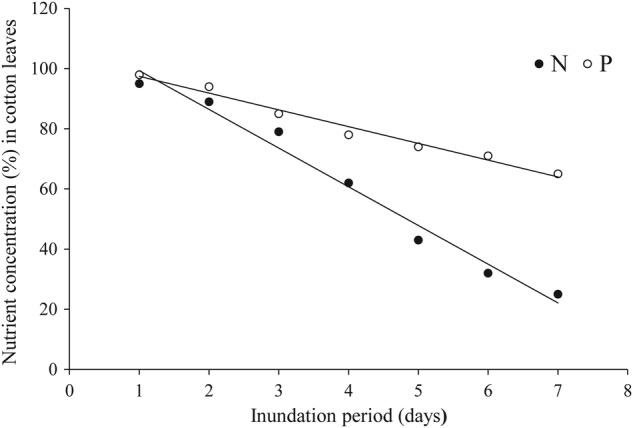
Changes in nutrient N (Hocking *et al*. 1985) and P (Hocking *et al*. 1987) status of cotton leaves under increasing inundation period (days) of water-table depth 40 cm.

In cotton, inhibition of nutrient uptake has been strongly correlated with the length of inundation period (Fig. 4), plant growth stage and soil fertility level (Hocking *et al*. 1985; Milroy *et al*. 2009). Waterlogging-induced inhibition of uptake and translocation of macro-nutrients (N, P and K) were pronounced during the period of high nutrient demand i.e. peak flowering (McLeod 2001). Hocking *et al*. (1987) also reported similar results after exposing cotton to 8 days of waterlogging during flowering. Likewise, in a comprehensive study on leaf nutrient dynamics of waterlogged cotton, Milroy *et al*. (2009) reported significant reduction in concentrations of most essential nutrients. They observed that nutrient concentrations in cotton leaves were relatively more sensitive to waterlogging during peak flowering compared with late reproductive stages.

Nutrient deficiency in leaves during reproductive growth could be ascribed to their role as a net nutrient exporter to fruits, especially from the late flowering growth phase (Rochester *et al*. 2012). Transport of nutrients towards developing fruits depletes the fixed pool of each nutrient element unless uptake rates through the roots can be sustained during an energy crisis. While inorganic nutrients are delivered to leaves through the xylem, redistribution to developing sinks such as fruits with low transpiration rates and high nutrient demand (Marschner and Marschner 2011) are achieved through the phloem. McLeod (2001) observed that waterlogging at peak flowering of cotton (96 DAS) reduced P and K, both mobile nutrients, in cotton tops (upper shoots) by 32 and 19 %, respectively. Consistent with the claim that nutrient redistribution is important during flooding events, nutrient deficits were more pronounced in leaves and stems compared with fruits.

In contrast to the essential mineral elements, soil waterlogging increases Na^+^ accumulation in sensitive plant species (Barrett-Lennard 2003). McLeod (2001) observed a significant increase in shoot Na concentration in waterlogged cotton leaves, where increased leaf Na concentrations were the result of higher Na translocation from roots to shoots rather than increased whole-plant uptake. Depolarization of hypoxic root plasma membranes does not diminish uptake of Na^+^ ions; indeed more Na^+^ ions enter via non-selective cation channels, while limited H^+^-ATPase activity impairs active Na^+^ exclusion across the plasma membrane and results in Na build up in root cells. Loading of anions and cations into the xylem requires various transporter channels (reviewed by Shabala and Mackay 2011). Although hypoxia blocks outwardly rectifying channels, Na^+^ enters the xylem via the non-selective outward-rectifying channels; hence the loss of selectivity for K^+^ over Na^+^ lies in contrasting uptake systems (Barrett-Lennard and Shabala 2013). Reduced retrieval of Na^+^ from the anoxic stele to the aerobic cortex might also be responsible for the relatively higher Na^+^ transport towards the shoot (Colmer and Greenway 2011).

### Yield

The effect of waterlogging on vegetative growth and yield of cotton depends on the cumulative time for which the root system remains under low soil O_2_ concentrations (O_2_ < 10 %), soil type and developmental stage (Milroy *et al*. 2009). Earlier studies showed that an inundation period of 4–32 h significantly limited cotton lint yield (Hodgson 1982). However, Bange *et al*. (2004) observed no significant impact on cotton yield after 72 h of waterlogging, suggesting that plant responses to waterlogging vary widely with experimental conditions. Improved performance during the recent years among waterlogged cotton crops has been attributed to better agronomic practices, reduction in soil compaction (a by-product of sustained waterlogging), use of modern technology for land levelling and the development of relatively waterlogging-tolerant cotton cultivars. Field studies have also confirmed that yield penalties in waterlogged cotton are strongly linked with ridge height; removing ridges exacerbated waterlogging damage while enhancing yield in aerobic conditions.

Waterlogging sensitivity in cotton is strongly associated with growth stage (McLeod 2001) but there is no *a priori* basis for temporal changes in tolerance. In a series of test-pit experiments, Wu *et al*. (2012) observed 27–30 % yield reduction after 4–9 days of waterlogging, respectively, during early reproductive stage in cotton. A 10-day exposure significantly increased young boll and square abscission in cotton, leading to a 42 % yield reduction (Jiang *et al*. 2013). Likewise, Bange *et al*. (2004) reported larger yield losses in cotton waterlogged at early squaring stage (65 DAS) compared with a later growth stage (112 DAS).

Higher waterlogging sensitivity during early reproductive growth in cotton has been notionally linked to the hormone-dependent shedding of young squares observed during abiotic stress (de Brito *et al*. 2013). Once established, the cotton bolls become less sensitive to stress-induced abscission. As the reduction in yield in waterlogged cotton crops is a function of lower fruiting number, fruit abscission after waterlogging has been directly implicated in yield losses (Bange *et al*. 2004). Waterlogging significantly suppressed plant growth and reproductive node development, reducing the total number of fruiting sites. Waterlogging-induced damage to cotton during later growth, as observed by McLeod (2001) in glasshouse experiments, was associated with inhibited nutrient uptake. However, with limited space for root growth and potential exhaustion of nutrients, nutrient deficiency was accentuated during peak boll development. Since the final yield was not recorded, it is not certain whether foliar nutrient deficiency translated into significant yield losses. Once formed, the cotton bolls become less sensitive to stress-induced abscission and may continue to be a nutrient sink by re-translocating nutrients from leaves.

## Physiological Processes and their Contribution to Waterlogging Damage

### Photosynthesis

**Figure 5.**
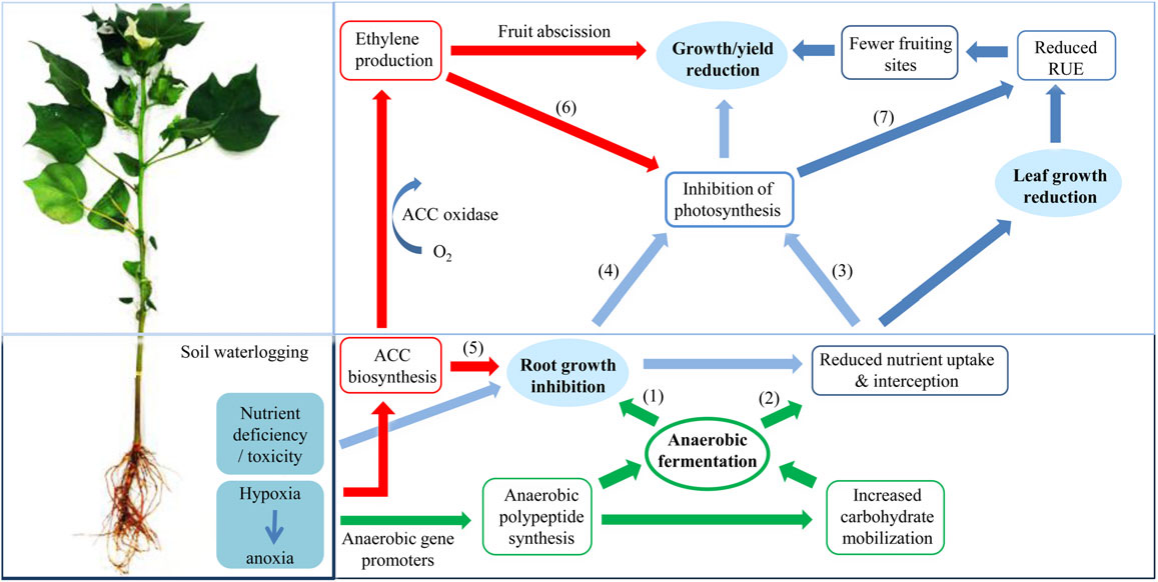
Changes in cotton growth and yield in response to soil waterlogging. Flows are represented in four categories: green (biochemical pathway); red (hormonal/signalling pathway); light blue (physiological pathways); dark blue (morphological changes). (1) Lower ATP synthesis under O_2_ deficiency inhibits root growth (Armstrong and Drew 2002). (2) Reduced plasma membrane H^+^-ATPase activity impairs nutrient uptake and interception (Jackson *et al*. 2003). (3) Limited nutrient transport to leaf tissues damage chlorophyll and photosynthesis (Meyer *et al*. 1987). (4) Inhibited root growth acts as a negative feedback to photosynthesis by reducing the root carbohydrate demand (Benjamin and Greenway 1979). (5) Higher ACC concentration in root tissues could inhibit root growth (Leblanc *et al*. 2008). (6) Ethylene can influence ABA-induced stomatal dynamic and photosynthesis (Else *et al*. 2009). (7) Inhibited leaf photosynthesis in turn influence biomass accumulation, leaf size, canopy development and overall radiation-use efficiency (Guang *et al*. 2012).

Flooding and subsequent soil waterlogging usually causes a rapid decline in photosynthetic rate, ranging from 10 to 90 % in different species (Kozlowski and Pallardy 1984). Various reasons for hypoxia-induced photosynthetic impairment are reported in the literature (Fig. 5). Waterlogging sensitivity of cotton has been strongly associated with photosynthetic inhibition (Najeeb *et al*. 2015*a*). In cotton, Milroy and Bange (2013) observed a significant drop in the rate of photosynthesis under sustained waterlogging treatments for 72 h, while rates recovered to normal as the soil O_2_ status improved. They showed that the rate of photosynthesis exhibited a degree of acclimation, becoming less responsive to soil O_2_ status during the later growth stages. Nutrient deficiency in cotton leaves has been considered the main reason for the fall in leaf photosynthetic rates. However, there was a lack of improvement in photosynthesis of waterlogged cotton under foliar and soil fertilizer (N, P and K) application (Meyer *et al*. 1987; Hodgson and MacLeod 1988; Ashraf *et al*. 2011; Zhou and Oosterhuis 2012) suggesting that long-distance signalling from roots might explain impaired leaf function; possibilities include hydraulics (e.g. stomatal closure) and hormones (e.g. changes in expression of critical photosynthetic genes, chlorophyll degradation).

Ahmed *et al*. (2006) suggested that early reduction in photosynthesis of waterlogged plants is regulated by internal damage to photosystem II (PSII) associated with photoinhibition, independent of stomatal closure. These non-stomatal/metabolic factors include intercellular gas diffusion, biochemical reactions, reduction in CO_2_ assimilation rates and quantum yield of PSII. Similarly, modification in synthesis, regulation and transport of endogenous hormones in cotton leaves influences photosynthetic CO_2_ fixation (Pandey *et al*. 2001). Down-regulation of sulfite reductase activity (a key enzyme of sulfate assimilation) could cause thylakoid damage and subsequent reduction in photosynthetic activity in waterlogged cotton leaves (Christianson *et al*. 2010*b*).

### Transpiration, stomatal behaviour and hormone physiology

At the inception of waterlogging, plant roots rapidly transmit xylem-borne signals to leaves in the form of hormones, most notably abscisic acid (ABA), slowing transpiration via stomatal closure (Jackson *et al*. 2003). Numerous studies reported stomatal closure and low transpiration rates in a range of plant species within hours up to days of waterlogging being imposed (Barrett-Lennard 2003; Mollard *et al*. 2008), although stomatal closure is not consistently reported for cotton. For example, some reports suggested that waterlogging reduces stomatal conductance and leaf water potential in cotton (Meyer *et al*. 1987; Christianson *et al*. 2010*a*), while Hocking *et al*. (1985) and McLeod (2001) observed no significant change in transpiration rate and stomatal conductance of waterlogged cotton. Likewise, Ashraf *et al*. (2011) found a significant reduction in leaf water potential without any significant change in leaf stomatal conductance, presumably due to impaired root hydraulics that occurs when roots are O_2_ deficient (Gibbs *et al*. 1998). Therefore, it is difficult to correlate growth inhibition in waterlogged cotton with perturbations to leaf water status. Effects on transpiration and stomatal conductance might be dependent on soil type, duration of waterlogging and plant growth stage, whereas photosynthesis responds more rapidly to O_2_ deficiency in root tissues. This uncoupling of water and carbon economies suggests that they are under independent controls when roots of cotton are waterlogged.

In sensitive plant species such as tomato and cotton, hypoxia-induced cytosolic acidosis causes conformational changes in root aquaporins, inhibiting water transport to leaves, thereby reducing turgor pressure in guard cells and closing stomata (Else *et al*. 2001; Hebbar and Mayee 2011). The similarity of the effects of waterlogging, exogenous ABA application (Else *et al*. 2009) and high external CO_2_ concentrations (Bradford 1983) on stomatal behaviour in tomato suggest a common mechanism, possibly with ABA as the key factor. The precise nature of root-derived ‘waterlogging’ signals remains unresolved (Else *et al*. 2009) and it is likely that specific signals operate in different time frames; short-term signalling could be achieved by loss of root hydraulic conductivity (Else *et al*. 2001) or increased 1-aminocyclopropane-1-carboxylic acid (ACC) transport (Bradford and Hsiao 1982), while ABA accumulation in leaves (Ahmed *et al*. 2006) might ensue more slowly, thus regulating stomatal conductance and photosynthesis and transpiration.

Hypoxic tomato roots release a large amount of ACC (the precursor to ethylene) into the transpiration stream due to inhibition of the oxidation of ACC and/or up-regulation of genes governing ACC synthesis (Bradford and Hsiao 1982). This ACC is converted into ethylene in the presence of O_2_ and ACC oxidase in the leaves. Elevated ethylene accumulation accelerates activity of an abscission layer in the peduncle, causing square and boll abscission and overall lint yield reduction in cotton (Lipe and Morgan 1973). Investigating responses of cotton to hypoxia, Christianson *et al*. (2010*b*) found increased expression of genes regulating ACC synthesis, pointing to the role of ethylene as a key signal in mediating responses to waterlogging. Higher ethylene accumulation accelerates generation of reactive oxygen species (ROS), which damage macromolecules and suppress photochemical efficiency (Ahmed *et al*. 2006), compromising organelles and ultimately causing cell death.

### Radiation-use efficiency

Crop growth rate depends on the amount of intercepted radiation and its concomitant conversion into biomass, which is termed radiation-use efficiency (RUE) (Monteith and Moss 1977). Leaf size and canopy architecture are major determinants of absorption of incoming photosynthetic active radiation, while conversion of intercepted radiation into new biomass mainly depends on the rate of net photosynthesis. However, the effect of other factors such as reproductive partitioning, growth conditions and plant developmental stage on RUE and crop growth rate cannot be overlooked (Passioura 1977). Therefore, integration of different physiological and growth processes is essential for estimating RUE or crop potential productivity.

Waterlogging suppresses leaf growth, canopy development and ultimately limits light interception in cotton (Guang *et al*. 2012). The growth reduction in waterlogged cotton was more strongly associated with low RUE than with the interception of light alone (Bange *et al*. 2004). There have been a number of reports illustrating negative impacts of waterlogging on RUE and lint yield of cotton through limiting dry matter production (Bange *et al*. 2004; Guang *et al*. 2012). Although a limited role of short-term shade incurs yield losses in severely waterlogged cotton, long-term shading can significantly increase damage (Najeeb *et al*. 2015*b*). Impaired nutrient uptake and translocation, especially N from waterlogged soils, seems responsible for impaired leaf growth (Milroy *et al*. 2009) and inhibition of photosynthesis. However, Milroy and Bange (2013) observed a weak association between photosynthetic rates and N contents of the youngest fully developed leaves of waterlogged cotton, and suggested uneven light distribution within the canopy might be responsible for low RUE of the whole plant. Since the value of RUE depends on the sum of photosynthetic performance through the whole canopy, collection of data (leaf N and photosynthesis) from the topmost leaves may not be adequate for estimating RUE under stressful environments. Exploring the effect of soil waterlogging on various canopy layers of cotton, Kuai *et al*. (2014) established that waterlogging more severely impaired chlorophyll pigments and consequently photosynthesis in the lower canopy leaves, while leaves at the top of canopy showed delayed damage by translocating nutrients from lower leaves. Thus variation in light penetration and nutrient distribution through the canopy should be considered when collecting data for leaf photosynthesis or nutrients.

## Metabolic Responses to Waterlogging in Crop Species

Rapid depletion of oxygen from the rhizosphere unbalances soil chemistry and disturbs energy and hormone metabolism, triggering the downstream physiological and biochemical events described in the previous section. Adaptive responses to these events are natural targets for improved waterlogging tolerance of cotton at the cell level. The known metabolic responses to oxygen deficit can be broadly divided into four groups:

- Induction of anaerobic polypeptides (ANPs), enabling carbohydrate mobilization and subsequent fermentation (Subbaiah and Sachs 2003);
- Regulation of intracellular pH and thereby, membrane charge, via changes in transporter activity. Acidosis determines activity of some key enzymes (e.g. pyruvate decarboxylase (PDC), nitrate reductase and nicotinamide adenine dinucleotide (NADH)-dependent glutamate synthase (Steffen *et al*. 2001)) and defines a new ‘set point’ for low-oxygen metabolism (Felle 2005);
- Alteration in expression pattern of genes controlling O_2_ sensing (Gibbs *et al*. 2011; Licausi *et al*. 2011). A recent publication by Mendiondo *et al*. (2015) illustrated that O_2_ sensing in barley is mediated by the N-end rule pathway. Sensing was achieved via an amino terminal cysteine residue *in vivo*, causing increased expression of hypoxia-associated genes and improved tolerance to waterlogging. Thus homologous components of the N-end rule pathway identified in barley are potential targets for engineering waterlogging tolerance in cotton. Similarly, activation of proteins regulating ROS signalling are potential targets for improved tolerance (Baxter-Burrell *et al*. 2002).
- Synthesis of non-symbiotic haemoglobin (nsHbs) proteins (Igamberdiev and Hill 2004; Sairam *et al*. 2009).

### Signalling pathways and gene regulation

Despite the improved understanding of responses to oxygen deficits brought about by proteomic and genomic approaches (Table 1), the full array of responses that can confer waterlogging tolerance remain elusive (Narsai *et al*. 2011). Microarray studies have consistently shown that hypoxia affects expression of genes coding for signal transduction (Baxter-Burrell *et al*. 2002), with sugar signalling in rice coleoptiles under anoxia (Lasanthi-Kudahettige *et al*. 2007) particularly likely to be relevant to diverse species during O_2_ deficits. Other examples of commonly observed gene expression responses in hypoxia involve ethylene biosynthesis, nitrogen metabolism and cell wall degeneration (Table 1). Up-regulation of common genes has been reported in O_2_ deficits across a wide range of plant taxa covering a spectrum of flood tolerance. These consistent changes suggest that evolutionary ‘solutions’ to surviving this most challenging of environmental stresses have their origins in ancient progenitors, often prokaryotic (Müller *et al*. 2001).

**Table 1.** Some commonly up and down-regulated processes, as identified by gene expression studies, when a range of higher plant species were exposed to low O_2_ conditions.

Species	Treatment	Genes up-regulated	Genes down-regulated	Reference
Cotton (*Gossypium hirsutum* L.)	Soil waterlogging	Glycolysis, fermentation and mitochondrial electron transport pathways, ethylene synthesis, alanine synthesis	Cell wall synthesis, flavonoid production and synthesis of amino acids	Christianson *et al*. (2010*b*)
*Arabidopsis*	Hypoxia (3 % oxygen)	Glycolysis, fermentation amino acid metabolism, ethylene synthesis, protein kinase activity, and auxin responses	Cell wall synthesis, nucleosome structures, water channels and ion transporters	Liu *et al*. (2005)
Poplar	Soil waterlogging	Glycolysis, fermentation, trehalose synthesis, proline synthesis	Signalling, phenylalanine synthesis	Christianson *et al*. (2010*a*)
Rice	Anoxia	Glycolysis, ethylene response factors, ethanolic fermentation, pyruvate metabolism, heat shock proteins, starch synthesis	PEP carboxylase, sugar transporters, catalase, signal transduction	Lasanthi-Kudahettige *et al*. (2007)
Sugar beet	Waterlogging	Glycolysis/pentose phosphate cycle, carbohydrate metabolism, seed specific proteins, transport, transcription, signal transduction, lipid metabolism, protein biosynthesis, protein folding, metabolism and cell division cycle	Cytoplasmic ribosomal proteins, translation initiation factors, seed storage proteins, late embryogenesis, seed maturation and dehydration proteins	Pestsova *et al*. (2008)
Maize	Submergence	Glycolysis, and ethanolic fermentation, auxin response factor, carbohydrate and energy metabolism	Starch synthase aminotransferase, homeostasis and signal cascades of hormone	Zhang *et al*. (2008)
Soybean	Submergence	Photosynthesis, glycolysis, Ser-Gly-Cys group amino acid synthesis, regulation of transcription, ubiquitin-mediated protein degradation and cell death	Synthesis of phosphofructokinase glucosyl and glucuronyl transferase, secondary metabolism, transport, cell wall synthesis, amino acid metabolism	Nanjo *et al*. (2011)

While transcriptomic responses to hypoxia and anoxia have common features across species (e.g. increased expression of genes for fermentative enzymes, sugar mobilization), transcriptomic profiles also bear characteristics of individual plant species (Christianson *et al*. 2010*a*; Narsai *et al*. 2011). Root tissues are the major target of hypoxic stress and could potentially regulate shoot responses (induction of hypoxia-responsive genes) via transport of metabolites such as γ-aminobutylate and alanine towards shoot (Mustroph *et al*. 2014). Analysis of carefully defined tissues from different organs (e.g. root apices, leaves) across a broad range of taxa is still called for, with datasets from these independent analyses of both transcriptomes and proteomes providing targets for identification of markers for hypoxia tolerance. Early transcriptomic contrasts in hypoxia-treated roots from cotton, *Arabidopsis* and grey poplar indicated that 4–10 % of all known genes were differentially expressed in response to hypoxia (Christianson *et al*. 2010*a*). In a microarray study of waterlogged cotton roots and leaves, Christianson *et al*. (2010*b*) observed up-regulation of genes controlling biochemical processes such as glycolysis, fermentation and mitochondrial electron transport pathways, again underlining the role of ethanolic fermentation and residual respiratory activity in plant survival under hypoxia. Down-regulation of genes could be an equally helpful insight into mechanisms of flood tolerance; examples include reduced expression of genes associated with the synthesis of cell walls, flavonoids and amino acids. We consider it important to distinguish those genes that are down-regulated as an inevitable result of lower growth rates (e.g. inhibited protein synthesis) from those that perform subtler regulatory roles such as in energy conservation (Atwell *et al*. 2015). Such distinctions can best be deduced in datasets from cereals where responses to flooding have been relatively well studied. Systematic analyses in cotton and particularly between wild and domestic cultivars would be invaluable in identifying scope for breeding programs.

### Metabolic adaptation

Waterlogging-tolerant plants may avoid O_2_ deficits through multifaceted cellular and organ level structural modifications. These processes are driven by phytohormones, with ethylene, gibberellins and abscisic acid having well-substantiated roles in cell-level responses to low O_2_. The past decade has seen a wider recognition for quiescence as a strategy for survival during submergence, conserving energy (Bailey-Serres and Voesenek 2008) during restricted O_2_ supply. This is the most likely route to improved waterlogging tolerance in field-grown cotton.

Alcoholic fermentation is the most important fermentative pathway in plants (Rees *et al*. 1987), during which pyruvate is first converted into acetaldehyde by PDC, and then into ethanol by alcohol dehydrogenase (ADH). Lehle *et al*. (1991) confirmed that ethanolic fermentation is the major metabolic pathway for energy generation in hypoxic cotton seeds. They exposed germinating seeds to moderate hypoxia (6–9 mmol O_2_ mol^−1^) and observed production rates of 439 and 10 nmol seed^−1^ h^−1^ ethanol and acetaldehyde, respectively. However, radicle growth was significantly reduced at these relatively low fermentation rates compared with tolerant plants (Table 2), indicating that cotton seeds generate insufficient energy from fermentation under waterlogging or that acetaldehyde toxicity impedes growth. This does not preclude engineering a higher level of fermentation in root apices or other tissues during waterlogging events. In an attempt to increase ethanolic fermentation and subsequent tolerance to O_2_ deficiency, Ellis *et al*. (2000) used transgenic cotton lines over-expressing ADH and PDC genes. Despite a significant increase (up to 80 %) in ethanol production in transgenic line, there was no significant increase in hypoxia or anoxia tolerance in terms of growth or plant survival, indicating that increased ethanol synthesis (and thus ATP synthesis) alone was not sufficient to confer tolerance. During the field studies, the same transgenic lines did not exhibit any improvement in yield of waterlogged or non-waterlogged cotton compared with their respective controls (Bange *et al*. 2010). Therefore, further biochemical and physiological studies are needed to determine the relationship between anaerobic fermentation and the capacity of cotton roots to survive under waterlogging in the field. It is likely that more components are involved in plant anoxia tolerance than just the few genes regulating fermentation rate. There are many possible candidates for proteins (e.g. pumps and enzymes in primary metabolism) that could be critical for survival of cotton tissues in anoxia; carbohydrate-mobilizing enzymes, ion transporters and ROS scavengers (Gill and Tuteja 2010) are some potential targets. Tolerance to toxic molecules such as acetaldehyde and metal ions also deserves attention.

**Table 2.** Variation in ethanol synthesis in different plant organs under oxygen deficit.

Species	Plant organ	Oxygen concentration	Ethanol synthesis rate	Reference
Cotton	Seeds	9 ± 4 mmol O_2_ mol^−1^	0.44 μmol h^−1^ seed^−1^	Lehle *et al*. (1991)
	Roots	Hypoxia (5 % O_2_)	0.05 μmol g^−1^ FW h^−1^	Ellis *et al*. (2000)
	Transgenic roots		0.06–0.1 μmol g^−1^ FW h^−1^	
Rice	Whole plant (14 days)	Anoxia (N_2_) 20 h	28 μmol g^−1^ FW h^−1^	Mustroph *et al*. (2006)
	Shoots (14 days)	Anoxia (N_2_) 4 h	50 μmol g^−1^ FW h^−1^	
	Roots (14 days)	Hypoxia (3 % O_2_)	2.5 μmol g^−1^ FW h^−1^	
	Coleoptiles	Anoxia (N_2_)	5.2–8.3 μmol g^−1^ FW h^−1^	Edwards *et al*. (2012)
			6.8–9.7 μmol g^−1^ FW h^−1^	
Maize	Root tips (3 days pre-hypoxic)	Anoxia (N_2_) 8 h	15.7 μmol g^−1^ FW h^−1^	Xia and Saglio (1992)
Lettuce	Roots (5 days)	Anoxia (N_2_) 6 h	1.8 μmol g^−1^ FW h^−1^	Kato-Noguchi (2000)
Wheat	Shoot (9 days)	Anoxia (N_2_) 4 h	1.1 μmol g^−1^ FW h^−1^	Mustroph *et al*. (2006)
	Roots (9 days)		1.3 μmol g^−1^ FW h^−1^	
*Arabidopsis*	Shoots	Hypoxia (5 % O_2_)	0.23 μmol g^−1^ FW h^−1^	Ismond *et al*. (2003)
	Roots (4 weeks)		0.04 μmol g^−1^ FW h^−1^	Tadege *et al*. (1998)
Tobacco	Root apex	Anoxia 4 h	0.04 μmol g^−1^ FW h^−1^	
	Root tissues (5–7 weeks)		4.5 μmol g^−1^ FW h^−1^	

### Anaerobic polypeptides—old and new candidates

Oxygen deficiency up-regulates the expression of a select group of genes that encode for stress tolerance pathways in plants (Baxter-Burrell *et al*. 2002). This set of proteins has been termed ANPs, although it should be emphasized that the exact composition of this group remains open to debate. Enzymes such as PDC, ADH and sucrose synthase (SuSy) are all critical for the breakdown of sucrose in glycolysis and subsequent fermentation (Subbaiah and Sachs 2003) and are undoubtedly ANPs. Variable numbers of what we define as ANPs are nominated for different plant species (Millar and Dennis 1996). The advent of modern technologies and informatics (e.g. sophisticated proteomics and RNA sequencing) will doubtless reveal new candidates for tolerance, including transcription factors (e.g. ethylene-responsive factors) and other regulatory molecules. Proteomic studies should be conducted in diverse cotton germplasm and waterlogging intensities in order to define the ANPs that characterize waterlogged root tissues.

### Other genetic improvement or selecting natural mutants

While ADH and PDC are essential fermentative enzymes that enable breakdown of sugars for energy production (Fig. 5), the supply of substrates is critical. Generation of phosphorylated sugars from sucrose via SuSy is an energetically favourable means of sustaining glycolysis (Huang *et al*. 2008) and supporting sucrose metabolism during post-stress recovery (Santaniello *et al*. 2014). Increased activity of SuSy is reported in root tissues of relatively anoxia-tolerant plant species such as rice and maize during anoxia, whereas lower tolerance to anoxia in *SuSy* knockout mutants of maize suggested a critical role of SuSy in energy conservation during O_2_ deficiency (Ricard *et al*. 1998). Invertases provide an alternative means of sucrose hydrolysis, releasing free monosaccharides at the cost of additional ATP for subsequent sugar phosphorylation. The relative contribution of these two mechanisms to sucrose breakdown deserves closer attention in waterlogging-intolerant species such as cotton.

Challenging a commonly held view that SuSy is the preferred pathway of sucrose breakdown to sustain glycolysis in low O_2_ conditions (Huang *et al*. 2008), Santaniello *et al*. (2014) suggested that both sucrose synthase and invertase play an important role in sucrose metabolism under O_2_ deficiency. No variation in ethanol production, energy status or waterlogging tolerance was observed between wild-type and *SuSy* knockout mutants. Sucrose metabolism is particularly important during periods of high-energy demand such as follows a flooding event, when anoxia-tolerant plants can augment ATP yield through a ‘Pasteur Effect’ by accelerating glycolysis (Gibbs and Greenway 2003). The capacity of roots to sustain substrate supply for a Pasteur Effect could be a goal for improved anoxia tolerance in cotton. An alarmingly rapid decline in expression of *SuSy* and *ADH* genes that regulate key catabolic and fermentative processes was observed in cotton roots within a short time after waterlogging (48–96 h), reflecting the poor tolerance of commercial *G. hirsutum* genotypes to hypoxia (Christianson *et al*. 2010*b*).

### Pyrophosphate

A possible role of pyrophosphate (PP_i_) as high-energy donor molecule that can substitute for some of the roles of ATP has been suggested in plants that survive O_2_ deficits (Carystinos *et al*. 1995; Atwell *et al*. 2015). Transcriptomic and proteomic studies indicated that anoxia activates a PP_i_-dependent step during energy metabolism, which directs scarce energy supplies to essential PPi-dependent reactions such SuSy, PP_i_-PFK, PPDK and a proton transporting vacuolar PP_i_ase in anoxia-tolerant species (Pestsova *et al*. 2008; Howell *et al*. 2009). A shift in the energy source from ATP to PP_i_ helps plants to meet their energy requirements and stabilizes membrane charge via solute transport and H^+^ pumping (Atwell *et al*. 2015). With relatively few genes involved in engineering improved anoxia tolerance via PP_i_ metabolism, and a precedent in rice where vacuolar PP_i_ase contributes to tolerance (Liu *et al*. 2010), this is an avenue that should be considered in cotton.

### Non-symbiotic haemoglobins (nsHbs)

Nitric oxide has been identified as a signalling molecule synthesized in plant and animal tissues in response to O_2_ deficiency (Igamberdiev and Hill 2004). If unregulated, NO and its precursor, nitrite, would cause functional damage to plants (Hill 2012). However, the realization that NO is part of an important signalling system and potentially energy transduction in plants has cast a new light on the importance of this molecule. In hypoxia-tolerant plants, cellular O_2_ deficiency up-regulates expression of the *Hb* genes *glb1* or *glb2* which leads to synthesis of nsHbs and scavenging of NO, ethylene and ROS (Zhao *et al*. 2008). Because nsHbs have such a high affinity for O_2_, in its oxidized form it can convert NO to nitrate and thereby drive a cycle that ultimately oxidizes NADH to NAD^+^ and supports ATP regeneration (Sairam *et al*. 2009). Increased expression of the nsHbs gene, *GhHb1*, is reported in cotton as a response to fungal attack (Qu *et al*. 2005), encouraging its application as a stress tolerance mechanism by detoxifying highly toxic NO and regulating cellular energy status.

## Strategies to Overcome Waterlogging Stress

Cotton cultivated on clay-rich, fine-textured soils often experiences poor drainage during flood or furrow irrigation and the situation becomes worse in poorly levelled fields and after rain events that cause soil waterlogging and O_2_ deficiency within hours to days under warm growing conditions. Recent advances in production systems have substantially improved productivity in cotton crops through appropriate field practices such as proper layout design, land levelling, increasing slope, scheduling irrigation and foliar fertilizers (Bange *et al*. 2004). Yield gains in commercial cotton crops in waterlogging-prone conditions rely upon these management practices although significant improvements in waterlogging tolerance could be made by exploiting genotype × management × environment interactions. Optimally, crop management practices should inform breeding for improved stress tolerance, drawing on new insights into mechanisms and increasing availability of genome sequences (Wang *et al*. 2012).

### Fertilizer application

Hypoxia-induced cotton growth and yield reduction could be the result of: (i) nutrient deficiency (Bange *et al*. 2004); (ii) increased ethylene accumulation (Christianson *et al*. 2010*b*) and/or (iii) impaired photosynthesis and net carbon fixation per unit of leaf area (RUE). Once the molecular O_2_ level in soil declines, depending on the intensity and duration of waterlogging, a series of chemical reactions takes place altering pH as well as nutrient status and availability in the soil (Kozlowski and Pallardy 1984; Rochester 2001).

If waterlogging depletes nutrient supply to plants, exogenous application of fertilizers could logically help the plants to recover from injury if nutrient ions can be made to enter a compromised root system. Therefore, nutrient species, application method, rate and timing should be considered to avoid the negative impact of nutrient imbalance on soil ecology and tissue toxicities (e.g. manganese). Incremental supplies of N to waterlogged cotton plants improved stomatal resistance, photosynthetic rate and growth (Goswami 1990). Guo *et al*. (2010) suggested that post-waterlogging N fertilization (240 kg ha^−1^) could contribute to waterlogging tolerance by improving root growth, vigour and photosynthesis in cotton.

Post-waterlogging fertilizer application has been suggested for ameliorating detrimental effects of hypoxia on growth and yield (Guo *et al*. 2010; Ashraf *et al*. 2011). Application of fertilizer during or just after waterlogging was less effective due to inefficient nutrient absorption capacity of impaired roots. Additionally, the applied N may become unavailable for plant uptake due to high leaching risks in the wet soils. Similarly, additional N applied at the late growth phase of cotton could cause excessive vegetative growth and harvesting problems. In essence, the response has to be aligned with the growth and yield that can be expected with the season remaining. Application of fertilizers 8 days after termination of waterlogging increased the recovery of cotton compared with the immediate post-waterlogging application (Li *et al*. 2013). Similarly, 5 days post-waterlogging application of additional 20–30 % fertilizer (above the normal rate) significantly increased the growth and yield of waterlogged cotton compared with unfertilized control plants (Wu *et al*. 2012). Hypoxia-induced damage to roots limits nutrient uptake from soil even if excessive nutrients are available, therefore, foliar fertilizer application is recommended for waterlogged plants. Effectiveness of foliar N has been established by Hodgson and MacLeod (1988), who found that pre-waterlogging foliar N application significantly ameliorated deleterious effects of waterlogging on cotton lint yield. A foliar spray of iron sulfate (FeSO_4_) prior to waterlogging ameliorated the negative effects of iron chlorosis, returning cotton foliage to its normal colour (Rochester 2001).

#### Role of anti-ethylene agents

Waterlogging-induced ethylene accumulation in cotton is associated with a wide range of injuries and stresses, and is responsible for young fruit abscission (Guinn 1982). Agents that inhibit the synthesis or perception of ethylene (e.g. aminoethoxyvinylglycine (AVG), aminoethoxycetic acid (AOA), 1-methylcyclopropene (1-MCP) and cobalt and silver ions) have been shown to control ethylene accumulation by blocking the biosynthetic pathway (Yang and Hoffman 1984) or ethylene action (McDaniel and Binder 2012).

Application of AVG and 1-MCP has been suggested to limit ethylene-induced damage in many crops (Hall and Smith 1995; Kawakami *et al*. 2010). Since early fruit shedding in stressed cotton is linked with higher ethylene accumulation, application of AVG is proposed to have potential for improving yield by limiting fruit abscission. Spraying variable doses of AVG (62.5, 125 g and 250 g [active ingredient] ha^−1^) just prior to waterlogging, Bange *et al*. (2010) showed improved boll number and seed cotton yield of waterlogged cotton. Similarly, positive role of 1-MCP has been investigated on water-stressed cotton plants, where it inhibited ethylene action and improved physiological processes such as stomatal resistance, water potential and antioxidant enzyme activity (Kawakami *et al*. 2010). In a 2-year field study, de Brito *et al*. (2013) recorded a positive effect of AVG and 1-MCP on cotton seed and lint yield. They suggested that AVG application during the initial reproductive phase is the best time for improving cotton yield both under stressed and unstressed conditions. In a recent study, Najeeb *et al*. (2015*a*) observed a negative correlation between ethylene production and cotton yield during waterlogging, suggesting that regulating ethylene production by AVG application can increase both photosynthesis and fruit retention of waterlogged cotton. In addition, we observed that eliminating ethylene sensitivity (via ethylene-insensitive cotton mutant) can significantly improve cotton performance under waterlogged as well as under non-waterlogged environments (U. Najeeb *et al*. Sydney University, unpubl. res.). As ethylene regulates lint production in cotton, engineering ethylene-insensitive plants could result in lower lint yield. Thus production of transgenic cotton plants with organ-specific ethylene sensitivity (in vegetative organs) may offer solution to this problem. This approach might have a broader application, with transgenic (ethylene-insensitive) plants enhancing abiotic stress tolerance in other plants (Grichko and Glick 2001; Sergeeva *et al*. 2006).

Combined application of fertilizer and growth regulators could be a better option for ameliorating waterlogged crops, as the fertilizers ensure nutrient supply, while growth regulators restrain physiological damage. However, only a few reports are available on application of plant growth regulators for inducing waterlogging tolerance in cotton, and further studies are needed to explore role of growth regulators for growth and yield improvement in waterlogged cotton. Post-waterlogging spray of urea (1 %) + potassium chloride (0.5 %) in combination with plant growth regulators [brassin (0.02 mg L^−1^) + diethyl aminoethyl hexanoate (10 mg L^−1^)] significantly increased growth and yield of waterlogged cotton (Li *et al*. 2013). Pre-waterlogging foliar ABA application increased tolerance to subsequent waterlogging-induced injury in cotton through improving leaf photosynthesis (Pandey *et al*. 2001). Improvements in weather forecasting signalling major rainfall events would assist in identifying the need to apply foliar fertilizers and hormones.

## Conclusions and Future Prospects

This review draws on our knowledge of the physiological and biochemical responses of plants to O_2_ limitation in order to understand how these processes affect growth and yield in cotton (Fig. 5). Waterlogging reduces nutrient availability, O_2_ diffusion and cellular respiration, which influence plant water relations and impair biomass gain. Yield losses are greatly exacerbated by developmental effects of waterlogging, including ethylene-induced abscission of flowers. The few field and glasshouse experiments conducted on waterlogged cotton plants reveal no singular explanation for growth and yield reduction, implying a need for deeper analysis of gene expression patterns and hormonal physiology. In particular, there is a still knowledge gap in our understanding of the genetic basis of adaptation to hypoxia in waterlogged soils, made more challenging by the narrow range of tolerance observed among cultivated cotton genotypes. The expression patterns of genes during short-term hypoxia may provide a clue to critical energy-transducing pathways that confer tolerance to transient floods. To improve waterlogging tolerance in the full lifecycle of a cotton crop, it will be necessary to identify the connection between environmental cues such as soil O_2_, light levels and temperature and gene expression (e.g. by promoter analysis), thereby identifying specific physiological and biochemical mechanisms that enable survival. Such information on the response of cotton plants to hypoxia and the post-stress recovery period will assist with conventional and transgenic breeding approaches to enhance waterlogging tolerance during both vegetative and reproductive development.

Earlier studies focussed on inducing waterlogging tolerance in cotton through fertilizer application, with less attention paid to manipulating hormone physiology. However, our data suggest that increased ethylene synthesis is responsible for fruit abscission and yield losses in waterlogged cotton and thus there is a need to explore the role of anti-ethylene agents to enhance waterlogging tolerance in cotton. Bioengineering could help to reduce ethylene accumulation by modifying the genes that regulate ACC biosynthesis or perception. These approaches could be highly effective in conjunction with sound crop management practices.

## Sources of Funding

Research included in this review was funded by the Cotton Research and Development Corporation (Australia) and Cruiser R&D fund from Syngenta Crop Protection Australia, Cotton Seed Distributors.

## Contributions by the Authors

Writing the initial draft, preparation of figures and tables and revision of drafts: U.N. Detailed revision of all drafts: B.J.A. Comments on later drafts: D.K.Y.T. and M.P.B.

## Conflict of Interest Statement

None declared.

## References

[PLV080C1] Ahmed S, Nawata E, Sakuratani T 2006 Changes of endogenous ABA and ACC, and their correlations to photosynthesis and water relations in mungbean (*Vigna radiata* (L.) Wilczak cv. KPS1) during waterlogging. Environmental and Experimental Botany 57:278–284. 10.1016/j.envexpbot.2005.06.006

[PLV080C2] Armstrong W, Beckett PM 1987 Internal aeration and development of stela anoxia in submerged roots. New Phytologist 105:221–245. 10.1111/j.1469-8137.1987.tb00860.x

[PLV080C3] Armstrong W, Drew MC 2002 Root growth and metabolism under oxygen deficiency. In: Waisel Y, Eshel A, Kafkaf U, eds. Plant roots: the hidden half. New York: Marcel Dekker.

[PLV080C4] Armstrong W, Webb T 1985 A critical oxygen pressure for root extension in rice. Journal of Experimental Botany 36:1573–1582. 10.1093/jxb/36.10.1573

[PLV080C5] Armstrong W, Webb T, Darwent M, Beckett PM 2009 Measuring and interpreting respiratory critical oxygen pressures in roots. Annals of Botany 103:281–293. 10.1093/aob/mcn177 18819952 PMC2707311

[PLV080C6] Ashraf MA, Ahmad MSA, Ashraf M, Al-Qurainy F, Ashraf MY 2011 Alleviation of waterlogging stress in upland cotton (*Gossypium hirsutum* L.) by exogenous application of potassium in soil and as a foliar spray. Crop and Pasture Science 62:25–38. 10.1071/CP09225

[PLV080C128] Atwell BJ, Kriedemann PE, Turnbull CGN (eds). 1999 Plants in Action. Melbourne: Macmillan Education Australia, 582.

[PLV080C7] Atwell BJ, Greenway H, Colmer TD 2015 Efficient use of energy in anoxia-tolerant plants with focus on germinating rice seedlings. New Phytologist 206:36–56. 25472708 10.1111/nph.13173

[PLV080C8] Bailey-Serres J, Colmer TD 2014 Plant tolerance of flooding stress–recent advances. Plant, Cell and Environment 37:2211–2215. 10.1111/pce.12420 25074340

[PLV080C9] Bailey-Serres J, Voesenek LACJ 2008 Flooding stress: acclimations and genetic diversity. Annual Review of Plant Biology 59:313–339. 10.1146/annurev.arplant.59.032607.092752 18444902

[PLV080C10] Bange M, Milroy S, Ellis M, Thongbai P 2010 Opportunities to reduce the impact of water-logging on cotton. In: Dove H, Culvenor RA, eds. Proceedings of 15th Agronomy Conference. Lincoln, New Zealand.

[PLV080C11] Bange MP, Milroy SP, Thongbai P 2004 Growth and yield of cotton in response to waterlogging. Field Crops Research 88:129–142. 10.1016/j.fcr.2003.12.002

[PLV080C12] Barrett-Lennard EG 2003 The interaction between waterlogging and salinity in higher plants: causes, consequences and implications. Plant and Soil 253:35–54. 10.1023/A:1024574622669

[PLV080C13] Barrett-Lennard EG, Shabala SN 2013 The waterlogging/salinity interaction in higher plants revisited–focusing on the hypoxia-induced disturbance to K^+^ homeostasis. Functional Plant Biology 40:872–882. 10.1071/FP12235 32481157

[PLV080C14] Baxter-Burrell A, Yang Z, Springer PS, Bailey-Serres J 2002 RopGAP4-dependent Rop GTPase rheostat control of *Arabidopsis* oxygen deprivation tolerance. Science 296:2026–2028. 10.1126/science.1071505 12065837

[PLV080C15] Benjamin LR, Greenway H 1979 Effects of a range of O_2_ concentrations on porosity of barley roots and on their sugar and protein concentrations. Annals of Botany 43:383–391.

[PLV080C16] Blokhina OB, Chirkova TV, Fagerstedt KV 2001 Anoxic stress leads to hydrogen peroxide formation in plant cells. Journal of Experimental Botany 52:1179–1190. 10.1093/jexbot/52.359.1179 11432936

[PLV080C17] Bradford KJ 1983 Effects of soil flooding on leaf gas exchange of tomato plants. Plant Physiology 73:475–479. 10.1104/pp.73.2.475 16663242 PMC1066487

[PLV080C18] Bradford KJ, Hsiao TC 1982 Stomatal behavior and water relations of waterlogged tomato plants. Plant Physiology 70:1508–1513. 10.1104/pp.70.5.1508 16662706 PMC1065914

[PLV080C19] Brändle R 1991 Flooding resistance of rhizomatous amphibious plants. In: Jackson MB, Davies DD, Lambers H, eds. Plant life under oxygen deprivation. Ecology, physiology and biochemistry. The Hague: SPB Academic, 35–46.

[PLV080C20] Carystinos GD, MacDonald HR, Monroy AF, Dhindsa RS, Poole RJ 1995 Vacuolar H(+)-translocating pyrophosphatase is induced by anoxia or chilling in seedlings of rice. Plant Physiology 108:641–649. 10.1104/pp.108.2.641 7610161 PMC157384

[PLV080C21] Christianson JA, Llewellyn DJ, Dennis ES, Wilson IW 2010a Comparisons of early transcriptome responses to low-oxygen environments in three dicotyledonous plant species. Plant Signaling and Behavior 5:1006–1009. 10.4161/psb.5.8.12231 20724824 PMC3115181

[PLV080C22] Christianson JA, Llewellyn DJ, Dennis ES, Wilson IW 2010b Global gene expression responses to waterlogging in roots and leaves of cotton (*Gossypium hirsutum* L.). Plant and Cell Physiology 51:21–37. 10.1093/pcp/pcp163 19923201

[PLV080C23] Colmer TD 2003 Long-distance transport of gases in plants: a perspective on internal aeration and radial oxygen loss from roots. Plant, Cell and Environment 26:17–36. 10.1046/j.1365-3040.2003.00846.x

[PLV080C24] Colmer TD, Greenway H 2011 Ion transport in seminal and adventitious roots of cereals during O_2_ deficiency. Journal of Experimental Botany 62:39–57. 10.1093/jxb/erq271 20847100

[PLV080C25] Conaty WC, Tan DKY, Constable GA, Sutton BG, Field DJ, Mamum EA 2008 Genetic variation for waterlogging tolerance in cotton. The Journal of Cotton Science 12:53–61.

[PLV080C26] Davies CL, Turner DW, Dracup M 2000 Yellow lupin (*Lupinus luteus*) tolerates waterlogging better than narrow-leafed lupin (*L. angustifolius*)—I. Shoot and root growth in a controlled environment. Australian Journal of Agricultural Research 51:701–709. 10.1071/AR99073

[PLV080C27] de Brito GG, de Barcellos Ferreira AC, Borin ALDC, de Lelis Morello C 2013 1-Methylcyclopropene and Aminoethoxyvinylglycine effects on yield components of field-grown cotton. Ciência e Agrotecnologia 37:9–16. 10.1590/S1413-70542013000100001

[PLV080C28] Dodd K, Guppy CN, Lockwood PV, Rochester IJ 2013 Impact of waterlogging on the nutrition of cotton (*Gossypium hirsutum* L.) produced in sodic soils. Crop and Pasture Science 64:816–824.

[PLV080C30] Edwards JM, Roberts TH, Atwell BJ 2012 Quantifying ATP turnover in anoxic coleoptiles of rice (*Oryza sativa*) demonstrates preferential allocation of energy to protein synthesis. Journal of Experimental Botany 63:4389–4402. 10.1093/jxb/ers114 22585748 PMC3421981

[PLV080C31] Ellis MH, Millar AA, Llewellyn DJ, Peacock WJ, Dennis ES 2000 Transgenic cotton (*Gossypium hirsutum*) over-expressing alcohol dehydrogenase shows increased ethanol fermentation but no increase in tolerance to oxygen deficiency. Australian Journal of Plant Physiology 27:1041–1050.

[PLV080C32] Else MA, Coupland D, Dutton L, Jackson MB 2001 Decreased root hydraulic conductivity reduces leaf water potential, initiates stomatal closure and slows leaf expansion in flooded plants of castor oil (*Ricinus communis*) despite diminished delivery of ABA from the roots to shoots in xylem sap. Physiologia Plantarum 111:46–54. 10.1034/j.1399-3054.2001.1110107.x

[PLV080C33] Else MA, Janowiak F, Atkinson CJ, Jackson MB 2009 Root signals and stomatal closure in relation to photosynthesis, chlorophyll a fluorescence and adventitious rooting of flooded tomato plants. Annals of Botany 103:313–323. 10.1093/aob/mcn208 19001430 PMC2707317

[PLV080C34] Evans DE 2004 Aerenchyma formation. New Phytologist 161:35–49. 10.1046/j.1469-8137.2003.00907.x

[PLV080C35] Felle HH 2005 pH regulation in anoxic plants. Annals of Botany 96:519–532. 10.1093/aob/mci207 16024558 PMC4247022

[PLV080C36] Gibbs DJ, Lee SC, Isa NM, Gramuglia S, Fukao T, Bassel GW, Correia CS, Corbineau F, Theodoulou FL, Bailey-Serres J, Holdsworth MJ 2011 Homeostatic response to hypoxia is regulated by the N-end rule pathway in plants. Nature 479:415–418. 10.1038/nature10534 22020279 PMC3223408

[PLV080C37] Gibbs J, Greenway H 2003 Mechanisms of anoxia tolerance in plants. I. Growth, survival and anaerobic catabolism. Functional Plant Biology 30:1–47. 10.1071/PP98095 32688990

[PLV080C38] Gibbs JJ, Turner DW, Armstrong W, Sivasithamparam K, Greenway H 1998 Response to oxygen deficiency in primary maize roots. II. Development of oxygen deficiency in the stele has limited short-term impact on radial hydraulic conductivity. Functional Plant Biology 25:759–763.

[PLV080C39] Gill SS, Tuteja N 2010 Reactive oxygen species and antioxidant machinery in abiotic stress tolerance in crop plants. Plant Physiology and Biochemistry 48:909–930. 10.1016/j.plaphy.2010.08.016 20870416

[PLV080C40] Goswami C 1990 Hormonal regulation of fertility-induced changes in stomatal diffusive resistance in waterlogged cotton (*Gossypium hirsutum* L.) var H-777. Indian Journal of Experimental Biology 28:585–587.

[PLV080C41] Grichko VP, Glick BR 2001 Flooding tolerance of transgenic tomato plants expressing the bacterial enzyme ACC deaminase controlled by the 35S, rolD or PRB-1b promoter. Plant Physiology and Biochemistry 39:19–25. 10.1016/S0981-9428(00)01217-1

[PLV080C42] Guang C, Xiugui W, Yu L, Wenbing L 2012 Effect of water logging stress on cotton leaf area index and yield. Procedia Engineering 28:202–209. 10.1016/j.proeng.2012.01.706

[PLV080C43] Guinn G 1982 Fruit age and changes in abscisic acid content, ethylene production, and abscission rate of cotton fruits. Plant Physiology 69:349–352. 10.1104/pp.69.2.349 16662207 PMC426208

[PLV080C44] Guo WQ, Chen BL, Liu RX, Zhou ZG 2010 Effects of nitrogen application rate on cotton leaf antioxidant enzyme activities and endogenous hormone contents under short-term waterlogging at flowering and boll-forming stage. Ying Yong Sheng Tai Xue Bao 21:53–60. 20387423

[PLV080C45] Hall MA, Smith AR 1995 Ethylene and the responses of plants to stress. Bulgarian Journal of Plant Physiology 21:71–79.

[PLV080C46] Hebbar KB, Mayee CD 2011 Parawilt/sudden wilt of cotton—a perspective on the cause and its management under field condition. Current Science 100:1654–1662.

[PLV080C47] Hill RD 2012 Non-symbiotic haemoglobins—What’s happening beyond nitric oxide scavenging? AoB PLANTS 2012:pls004; 10.1093/aobpla/pls004. PMC3292739 22479675

[PLV080C48] Hocking PJ, Reicosky DC, Meyer WS 1985 Nitrogen status of cotton subjected to two short term periods of waterlogging of varying severity using a sloping plot water-table facility. Plant and Soil 87:375–391. 10.1007/BF02181905

[PLV080C49] Hocking PJ, Reicosky DC, Meyer WS 1987 Effects of intermittent waterlogging on the mineral nutrition of cotton. Plant and Soil 101:211–221. 10.1007/BF02370647

[PLV080C50] Hodgson AS 1982 The effects of duration, timing and chemical amelioration of short-term waterlogging during furrow irrigation of cotton in a cracking grey clay. Australian Journal of Agricultural Research 33:1019–1028. 10.1071/AR9821019

[PLV080C51] Hodgson AS, MacLeod DA 1988 Seasonal and soil fertility effects on the response of waterlogged cotton to foliar-applied nitrogen fertilizer. Agronomy Journal 80:259–265. 10.2134/agronj1988.00021962008000020021x

[PLV080C52] Howell KA, Narsai R, Carroll A, Ivanova A, Lohse M, Usadel B, Millar AH, Whelan J 2009 Mapping metabolic and transcript temporal switches during germination in rice highlights specific transcription factors and the role of RNA instability in the germination process. Plant Physiology 149:961–980. 10.1104/pp.108.129874 19074628 PMC2633829

[PLV080C53] Huang S, Colmer TD, Millar AH 2008 Does anoxia tolerance involve altering the energy currency towards PPi? Trends in Plant Science 13:221–227. 10.1016/j.tplants.2008.02.007 18439868

[PLV080C54] Huck MG 1970 Variation in taproot elongation rate as influenced by composition of the soil air. Agronomy Journal 62:815–818. 10.2134/agronj1970.00021962006200060042x

[PLV080C55] Igamberdiev AU, Hill RD 2004 Nitrate, NO and haemoglobin in plant adaptation to hypoxia: an alternative to classic fermentation pathways. Journal of Experimental Botany 55:2473–2482. 10.1093/jxb/erh272 15448180

[PLV080C56] International Cotton Advisory Committee on Cotton Yields—ICAC. 2009 A report by the Technical Information Section of the International Cotton Advisory Committee. Washington, DC.

[PLV080C57] Ismond KP, Dolferus R, De Pauw M, Dennis ES, Good AG 2003 Enhanced low oxygen survival in *Arabidopsis* through increased metabolic flux in the fermentative pathway. Plant Physiology 132:1292–1302. 10.1104/pp.103.022244 12857811 PMC167069

[PLV080C58] Jackson MB, Drew MC 1984 Effects of flooding on growth and metabolism of herbaceous plants. In: Kozlowski TT, ed. Flooding and plant growth. New York: Academic Press.

[PLV080C59] Jackson MB, Saker LR, Crisp CM, Else MA, Janowiak F 2003 Ionic and pH signalling from roots to shoots of flooded tomato plants in relation to stomatal closure. Plant and Soil 253:103–113. 10.1023/A:1024588532535

[PLV080C60] Jenkins JN 2003 Cotton. In: Traditional crop breeding practices: an historical review to serve as a baseline for assessing the role of modern biotechnology. Paris: OECD, 61–70.

[PLV080C61] Jiang Z-H, Zhua J-Q, Yang W, Lia M-F, Yua Y 2013 Effects of remedial measures implemented after waterlogging on cotton. In: Third International Conference on Intelligent System Design and Engineering Applications (ISDEA) Hong Kong: IEEE, 692–695.

[PLV080C62] Kato-Noguchi H 2000 Evaluation of the importance of lactate for the activation of ethanolic fermentation in lettuce roots in anoxia. Physiologia Plantarum 109:28–33. 10.1034/j.1399-3054.2000.100105.x

[PLV080C63] Kawakami EM, Oosterhuis DM, Snider JL 2010 Physiological effects of 1-Methylcyclopropene on well-watered and water-stressed cotton plants. Journal of Plant Growth Regulation 29:280–288. 10.1007/s00344-009-9134-3

[PLV080C64] Kozlowski TT, Pallardy SG 1984 Effect of flooding on water, carbohydrate and mineral relations. In: Kozlowski TT, ed. Flooding and plant growth. London: Academic Press.

[PLV080C65] Kuai J, Liu Z, Wang Y, Meng Y, Chen B, Zhao W, Zhou Z, Oosterhuis DM 2014 Waterlogging during flowering and boll forming stages affects sucrose metabolism in the leaves subtending the cotton boll and its relationship with boll weight. Plant Science 223:79–98. 10.1016/j.plantsci.2014.03.010 24767118

[PLV080C66] Lasanthi-Kudahettige R, Magneschi L, Loreti E, Gonzali S, Licausi F, Novi G, Beretta O, Vitulli F, Alpi A, Perata P 2007 Transcript profiling of the anoxic rice coleoptile. Plant Physiology 144:218–231. 10.1104/pp.106.093997 17369434 PMC1913783

[PLV080C67] Leblanc A, Renault H, Lecourt J, Etienne P, Deleu C, Le Deunff E 2008 Elongation changes of exploratory and root hair systems induced by aminocyclopropane carboxylic acid and aminoethoxyvinylglycine affect nitrate uptake and BnNrt2.1 and BnNrt1.1 transporter gene expression in oilseed rape. Plant Physiology 146:1928–1940. 10.1104/pp.107.109363 18287493 PMC2287360

[PLV080C68] Lehle FR, Zegeer AM, Ahmed OK 1991 Ethanolic fermentation in hypoxic cotton seed. Crop Science 31:746–750. 10.2135/cropsci1991.0011183X003100030042x

[PLV080C69] Li M-F, Zhu J-Q, Jiang Z-H 2013 Plant growth regulators and nutrition applied to cotton after waterlogging. In: Third International Conference on Intelligent System Design and Engineering Applications (ISDEA) Hong Kong: IEEE.

[PLV080C70] Licausi F, Kosmacz M, Weits DA, Giuntoli B, Giorgi FM, Voesenek LACJ, Perata P, van Dongen JT 2011 Oxygen sensing in plants is mediated by an N-end rule pathway for protein destabilization. Nature 479:419–422. 10.1038/nature10536 22020282

[PLV080C71] Lipe JA, Morgan PW 1973 Location of ethylene production in cotton flowers and dehiscing fruits. Planta 115:93–96. 10.1007/BF00388610 24458822

[PLV080C72] Liu F, VanToai T, Moy LP, Bock G, Linford LD, Quackenbush J 2005 Global transcription profiling reveals comprehensive insights into hypoxic response in *Arabidopsis*. Plant Physiology 137:1115–1129. 10.1104/pp.104.055475 15734912 PMC1065411

[PLV080C73] Liu Q, Zhang Q, Burton RA, Shirley NJ, Atwell BJ 2010 Expression of vacuolar H^+^-pyrophosphatase (OVP3) is under control of an anoxia-inducible promoter in rice. Plant Molecular Biology 72:47–60. 19763843 10.1007/s11103-009-9549-z

[PLV080C74] Marschner H, Marschner P 2011 In: Marschner P, ed. Marschner's mineral nutrition of higher plants. 3rd edn. Amsterdam, Netherlands: Elsevier/Academic Press.

[PLV080C75] McDaniel BK, Binder BM 2012 Ethylene receptor 1 (ETR1) is sufficient and has the predominant role in mediating inhibition of ethylene responses by silver in *Arabidopsis thaliana*. Journal of Biological Chemistry 287:26094–26103. 10.1074/jbc.M112.383034 22692214 PMC3406693

[PLV080C76] McLeod IG 2001 The effect of waterlogging and ion interactions on the development of premature senescence in irrigated cotton. PhD Thesis, the University of New England, Australia.

[PLV080C77] Mendiondo GM, Gibbs DJ, Szurman-Zubrzycka M, Korn A, Marquez J, Szarejko I, Maluszynski M, King J, Axcell B, Smart K, Corbineau F, Holdsworth MJ 2015 Enhanced waterlogging tolerance in barley by manipulation of expression of the N-end rule pathway E3 ligase PROTEOLYSIS6. Plant Biotechnology Journal; 10.1111/pbi.12334. PMC5098238 25657015

[PLV080C78] Meyer WS, Reicosky DC, Barrs HD, Smith RCG 1987 Physiological responses of cotton to a single waterlogging at high and low N-levels. Plant and Soil 102:161–170. 10.1007/BF02370698

[PLV080C79] Millar AA, Dennis ES 1996 Protein synthesis during oxygen deprivation in cotton. Functional Plant Biology 23:341–348.

[PLV080C80] Milroy SP, Bange MP 2013 Reduction in radiation use efficiency of cotton (*Gossypium hirsutum* L.) under repeated transient waterlogging in the field. Field Crops Research 140:51–58. 10.1016/j.fcr.2012.10.016

[PLV080C81] Milroy SP, Bange MP, Thongbai P 2009 Cotton leaf nutrient concentrations in response to waterlogging under field conditions. Field Crops Research 113:246–255. 10.1016/j.fcr.2009.05.012

[PLV080C82] Mollard FPO, Striker GG, Ploschuk EL, Vega AS, Insausti P 2008 Flooding tolerance of *Paspalum dilatatum* (Poaceae: Paniceae) from upland and lowland positions in a natural grassland. Flora—Morphology, Distribution, Functional Ecology of Plants 203:548–556. 10.1016/j.flora.2007.10.003

[PLV080C83] Monteith JL, Moss CJ 1977 Climate and the efficiency of crop production in Britain [and discussion]. Philosophical Transactions of the Royal Society B: Biological Sciences 281:277–294. 10.1098/rstb.1977.0140

[PLV080C84] Müller M, Lee JA, Gordon P, Gaasterland T, Sensen CW 2001 Presence of prokaryotic and eukaryotic species in all subgroups of the PPi-dependent group II phosphofructokinase protein family. Journal of Bacteriology 183:6714–6716. 10.1128/JB.183.22.6714-6716.2001 11673446 PMC95507

[PLV080C85] Mustroph A, Boamfa EI, Laarhoven LJJ, Harren FJM, Albrecht G, Grimm B 2006 Organ-specific analysis of the anaerobic primary metabolism in rice and wheat seedlings. I: dark ethanol production is dominated by the shoots. Planta 225:103–114. 10.1007/s00425-006-0333-x 16845530

[PLV080C86] Mustroph A, Kaiser KA, Larive CK, Bailey-Serres J 2014 Characterization of distinct root and shoot responses to low-oxygen stress in *Arabidopsis* with a focus on primary C-and N-metabolism. Plant, Cell and Environment 37:2366–2380. 10.1111/pce.12282 24450922

[PLV080C87] Najeeb U, Atwell BJ, Bange MP, Tan DKY 2015a Aminoethoxyvinylglycine (AVG) ameliorates waterlogging-induced damage in cotton by inhibiting ethylene synthesis and sustaining photosynthetic capacity. Plant Growth Regulation 76:83–98. 10.1007/s10725-015-0037-y

[PLV080C88] Najeeb U, Bange MP, Atwell BJ, Tan DKY 2015b Understanding the interactive effect of waterlogging and shade on cotton (*Gossypium hirsutum* L.) growth and yield. In: Proccedings of Agriculture and Climate Change conference “Adopting Crops To Increased Uncertainty”, Amsterdam, The Netherlands.

[PLV080C89] Nanjo Y, Maruyama K, Yasue H, Yamaguchi-Shinozaki K, Shinozaki K, Komatsu S 2011 Transcriptional responses to flooding stress in roots including hypocotyl of soybean seedlings. Plant Molecular Biology 77:129–144. 10.1007/s11103-011-9799-4 21656040

[PLV080C90] Narsai R, Rocha M, Geigenberger P, Whelan J, van Dongen JT 2011 Comparative analysis between plant species of transcriptional and metabolic responses to hypoxia. New Phytologist 190:472–487. 10.1111/j.1469-8137.2010.03589.x 21244431

[PLV080C91] Pandey DM, Goswami CL, Kumar B, Jain S 2001 Hormonal regulation of photosynthetic enzymes in cotton under water stress. Photosynthetica 38:403–407. 10.1023/A:1010925604941

[PLV080C92] Pang J, Shabala S 2010 Membrane transporters and waterlogging tolerance. In: Waterlogging signalling and tolerance in plants. Berlin: Springer, 197–219.

[PLV080C93] Passioura J 1977 Grain yield, harvest index, and water use of wheat. Journal of the Australian Institute of Agricultural Science 43:117–120.

[PLV080C94] Pestsova E, Meinhard J, Menze A, Fischer U, Windhovel A, Westhoff P 2008 Transcript profiles uncover temporal and stress-induced changes of metabolic pathways in germinating sugar beet seeds. BMC Plant Biology 8:122 10.1186/1471-2229-8-122 19046420 PMC2632670

[PLV080C95] Qu Z-L, Wang H-Y, Xia G-X 2005 GhHb1: a nonsymbiotic hemoglobin gene of cotton responsive to infection by *Verticillium dahliae*. Biochimica et Biophysica Acta (BBA)—Gene Structure and Expression 1730:103–113. 10.1016/j.bbaexp.2005.06.009 16084605

[PLV080C96] Rees T, Jenkin L, Smith A, Wilson P, Crawford R 1987 The metabolism of flood-tolerant plants. In: Crawford RMM, Spence DHN, eds. Plant life in aquatic and amphibious habitats. Oxford: Blackwell Scientific Publications, 227–238.

[PLV080C97] Ricard B, Van Toai T, Chourey P, Saglio P 1998 Evidence for the critical role of sucrose synthase for anoxic tolerance of maize roots using a double mutant. Plant Physiology 116:1323–1331. 10.1104/pp.116.4.1323 9536049 PMC35039

[PLV080C98] Rochester I 2001 Nutripak: a practical guide to cotton nutrition. Australian Cotton Cooperative Research Centre, ed. Narrabri: CSIRO Publishing.

[PLV080C99] Rochester IJ, Constable GA, Oosterhuis DM, Errington M 2012 Nutritional requirements of cotton during flowering and fruiting. Flowering and Fruiting in Cotton 35–45.

[PLV080C100] Saglio PH 1985 Effect of path or sink anoxia on sugar translocation in roots of maize seedlings. Plant Physiology 77:285–290. 10.1104/pp.77.2.285 16664043 PMC1064504

[PLV080C101] Sairam RK, Kumutha D, Ezhilmathi K 2009 Waterlogging tolerance: nonsymbiotic haemoglobin-nitric oxide homeostasis and antioxidants. Current Science 96:674–682.

[PLV080C102] Santaniello A, Loreti E, Gonzali S, Novi G, Perata P 2014 A reassessment of the role of sucrose synthase in the hypoxic sucrose-ethanol transition in *Arabidopsis*. Plant, Cell and Environment 37:2294–2302. 10.1111/pce.12363 24810896

[PLV080C103] Sergeeva E, Shah S, Glick BR 2006 Growth of transgenic canola (*Brassica napus* cv. Westar) expressing a bacterial 1-aminocyclopropane-1-carboxylate (ACC) deaminase gene on high concentrations of salt. World Journal of Microbiology and Biotechnology 22:277–282. 10.1007/s11274-005-9032-1

[PLV080C104] Setter TL, Waters I 2003 Review of prospects for germplasm improvement for waterlogging tolerance in wheat, barley and oats. Plant and Soil 253:1–34. 10.1023/A:1024573305997

[PLV080C105] Setter TL, Waters I, Sharma SK, Singh KN, Kulshreshtha N, Yaduvanshi NPS, Ram PC, Singh BN, Rane J, McDonald G, Khabaz-Saberi H, Biddulph TB, Wilson R, Barclay I, McLean R, Cakir M 2009 Review of wheat improvement for waterlogging tolerance in Australia and India: the importance of anaerobiosis and element toxicities associated with different soils. Annals of Botany 103:221–235. 10.1093/aob/mcn137 18708642 PMC2707304

[PLV080C106] Shabala S, Mackay A 2011 Ion transport in halophytes. In: Kader J, Delseny M, eds. Advances in botanical research, Vol. 57 Burlington: Elsevier Ltd, Academic Press, 151–187.

[PLV080C107] Shabala S, Shabala L, Barcelo J, Poschenrieder C 2014 Membrane transporters mediating root signalling and adaptive responses to oxygen deprivation and soil flooding. Plant, Cell and Environment 37:2216–2233. 10.1111/pce.12339 24689809

[PLV080C108] Smith CW 1995 Cotton (*Gossypium hirsutum* L.). Chapter 6. In: Crop production: evolution, history, and technology. New York: John Wiley and Sons, Inc., 287–349.

[PLV080C109] Steffen D, Döring O, Busch MA, Böttger M, Lüthje S 2001 Interaction between electron transport at the plasma membrane and nitrate uptake by maize (*Zea mays* L.) roots. Protoplasma 217:70–76. 10.1007/BF01289416 11732341

[PLV080C110] Steffens D, Hutsch B, Eschholz T, Losak T, Schubert S 2005 Water logging may inhibit plant growth primarily by nutrient deficiency rather than nutrient toxicity. Plant, Soil and Environment 51:545.

[PLV080C111] Stewart JM 1995 Potential for crop improvement with exotic germplasm and genetic engineering. In: Constable GA, Forrester NW, eds. Melbourne, Australia: CSIRO, 313–327.

[PLV080C112] Subbaiah CC, Sachs MM 2003 Calcium-mediated responses of maize to oxygen deprivation. Russian Journal of Plant Physiology 50:752–761. 10.1023/B:RUPP.0000003273.44823.cd

[PLV080C113] Tadege M, Brändle R, Kuhlemeier C 1998 Anoxia tolerance in tobacco roots: effect of overexpression of pyruvate decarboxylase. The Plant Journal 14:327–335. 10.1046/j.1365-313X.1998.00130.x

[PLV080C114] Teakle N, Flowers T, Real D, Colmer T 2007 Lotus tenuis tolerates the interactive effects of salinity and waterlogging by ‘excluding’ Na^+^ and Cl^−^ from the xylem. Journal of Experimental Botany 58:2169–2180. 10.1093/jxb/erm102 17510213

[PLV080C115] USDA. 2012 World Agricultural Production. United States Department of Agriculture, Foreign Agricultural Service. Circular Series WAP 8–15 August 2015.

[PLV080C116] Verstraeten I, Schotte S, Geelen D 2014 Hypocotyl adventitious root organogenesis differs from lateral root development. Frontiers in Plant Science; 10.3389/fpls.2014.00495. PMC4179338 25324849

[PLV080C117] Wang K, Wang Z, Li F, Ye W, Wang J, Song G, Yue Z, Cong L, Shang H, Zhu S, Zou C, Li Q, Yuan Y, Lu C, Wei H, Gou C, Zheng Z, Yin Y, Zhang X, Liu K, Wang B, Song C, Shi N, Kohel RJ, Percy RG, Yu JZ, Zhu YX, Wang J, Yu S 2012 The draft genome of a diploid cotton *Gossypium raimondii*. Nature Genetics 44:1098–1103. 10.1038/ng.2371 22922876

[PLV080C118] Wendel JF 1989 New World tetraploid cottons contain Old World cytoplasm. Proceedings of the National Academy of Sciences of the USA 86:4132–4136. 10.1073/pnas.86.11.4132 16594050 PMC287403

[PLV080C119] Wu QX, Zhu JQ, Liu KW, Guo CL 2012 Effects of fertilization on growth and yield of cotton after surface waterlogging elimination. Advance Journal of Food Science and Technology 4:398–403.

[PLV080C120] Wu Z, Soliman KM, Zipf A, Saha S, Sharma GC, Jenkins JN 2005 Isolation and characterization of genes differentially expressed in fiber of *Gossypium barbadense* L. The Journal of Cotton Science 9:166–174.

[PLV080C121] Xia JH, Saglio PH 1992 Lactic acid efflux as a mechanism of hypoxic acclimation of maize root tips to anoxia. Plant Physiology 100:40–46. 10.1104/pp.100.1.40 16652975 PMC1075514

[PLV080C122] Yang SF, Hoffman NE 1984 Ethylene biosynthesis and its regulation in higher plants. Annual Review of Plant Physiology 35:155–189. 10.1146/annurev.pp.35.060184.001103

[PLV080C123] Zeng F, Shabala L, Zhou M, Zhang G, Shabala S 2013 Barley responses to combined waterlogging and salinity stress: separating effects of oxygen deprivation and elemental toxicity. Frontiers in Plant Science 4:313 10.3389/fpls.2013.00313 23967003 PMC3743405

[PLV080C124] Zhang Z, Wei L, Zou X, Tao Y, Liu Z, Zheng Y 2008 Submergence-responsive microRNAs are potentially involved in the regulation of morphological and metabolic adaptations in maize root cells. Annals of Botany 102:509–519. 10.1093/aob/mcn129 18669574 PMC2701776

[PLV080C125] Zhang Y, Song X, Yang G, Li Z, Lu H, Kong X, Eneji AE, Dong H 2015 Physiological and molecular adjustment of cotton to waterlogging at peak-flowering in relation to growth and yield. Field Crops Research 179:164–172. 10.1016/j.fcr.2015.05.001

[PLV080C126] Zhao L, Gu R, Gao P, Wang G 2008 A nonsymbiotic hemoglobin gene from maize, ZmHb, is involved in response to submergence, high-salt and osmotic stresses. Plant Cell, Tissue and Organ Culture 95:227–237. 10.1007/s11240-008-9436-3

[PLV080C127] Zhou Z, Oosterhuis DM 2012 Physiological mechanism of nitrogen mediating cotton (*Gossypium hirsutum* L.) seedlings growth under water-stress conditions. American Journal of Plant Sciences 3:721–730. 10.4236/ajps.2012.36087

